# Liraglutide-Induced Hemorrhagic Pancreatitis in a Nondiabetic Patient

**DOI:** 10.14309/crj.0000000000000380

**Published:** 2020-05-06

**Authors:** Russell D. Dolan, Ahmad Najdat Bazarbashi, Amy Lo, Benjamin N. Smith

**Affiliations:** 1 Division of Gastroenterology, Hepatology and Endoscopy, Brigham and Women's Hospital, Harvard Medical School, Boston, MA

## Abstract

Liraglutide is a glucagon-like peptide 1 (GLP-1) analog with a primary effect of increased glucose-dependent insulin secretion and decreased gastric emptying. It serves as a feasible medical weight loss option because of the drug's effect of invoking satiety and lowering caloric intake. Despite emerging efficacy, the GLP-1 class has been associated with severe, although rare, adverse events, including pancreatitis. Previous investigations demonstrated the greatest association between GLP-1 agonist-associated pancreatitis in diabetic populations; however, liraglutide dosing is higher in weight loss formulations, placing the weight loss population at higher risk. We present a case of liraglutide-induced acute pancreatitis in a patient without diabetes.

## INTRODUCTION

Liraglutide is a glucagon-like peptide 1 (GLP-1) analog with a primary effect of increased glucose-dependent insulin secretion and decreased gastric emptying. It has been well-established to lower hemoglobin A1C and invoke satiety, lowering caloric intake, and therefore assisting with medical weight loss.

Despite emerging evidence of efficacy, the GLP-1 medication class has been associated with adverse reactions in animal models, including that of acute pancreatitis.^1^ This has been shown to be a result of ductal replication and acinar inflammation within the pancreas in rodent models.^2^ Furthermore, there has been evidence suggesting an association between GLP-1 agonists and acute pancreatitis in humans; however, this has predominantly been demonstrated within the diabetic population.^3,4^ We describe a nondiabetic patient who presented with abdominal pain and was found to have liraglutide-induced hemorrhagic pancreatitis.

## CASE REPORT

A 31-year-old woman with a history of morbid obesity (body mass index 40 kg/m^2^) on liraglutide for weight loss as bridge to bariatric surgery, cholelithiasis complicated by acute cholecystitis with a history of cholecystectomy 3 years before, hypothyroidism, fatty liver, and recent admission for “idiopathic” pancreatitis 1-month ago. She presented with sharp midepigastric pain radiating to her back and left upper abdomen. She endorsed nausea and nonbloody emesis; her symptoms were unrelated to food, and she was without other alleviating/aggravating factors. The pain was similar in character; however, it was more intense than her recent episode of acute pancreatitis.

She underwent extensive evaluation during her previous presentation of acute pancreatitis. Laboratory test results revealed normal complete blood count and comprehensive metabolic panel (including hepatic function panel); her lipase was 156. Abdominal and pelvic computed tomography (CT) revealed mild interstitial pancreatitis, but with a normal common bile duct without stones. Given normal liver enzymes and biliary appearance on CT, a magnetic resonance cholangiopancreatography was not performed. Additional laboratory test results revealed mildly elevated triglycerides (279 mg/dL), and her hemoglobin A1C was 6.0 with normal calcium, thyroid-stimulating hormone, and immunoglobulin G4 levels. She had no history of significant alcohol intake or tobacco exposure, and her urine drug screen was unremarkable. She denied a recent history of medication changes and abstained from nonsteroidal anti-inflammatory drugs; however, she had been maintained on Saxenda (liraglutide) 3 mg once daily for a total of 10 months for weight loss during preprocedural planning for bariatric surgery. This medication was discontinued at admission during her previous episode of pancreatitis. Her sole medication before the current presentation was levothyroxine 75 μg once daily for hypothyroidism. She was managed conservatively and discharged several days later.

**Figure 1.**
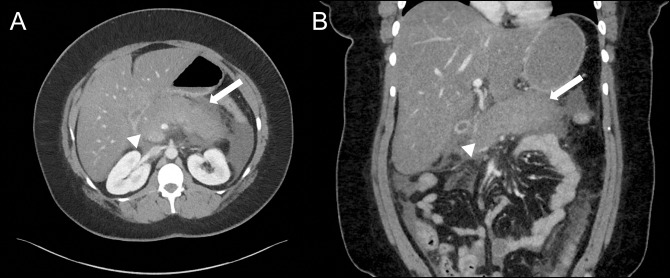
Abdominal and pelvic computed tomography with intravenous contrast in (A) axial and (B) coronal views showing the pancreas appearing diffusely edematous with surrounding stranding most severely affecting the body and tail with peripancreatic stranding/fluid (arrows) and sparing of pancreatic head (arrowheads). Biliary tree appears normal without choledocholithiasis and pancreatic duct is nondilated.

**Figure 2.**
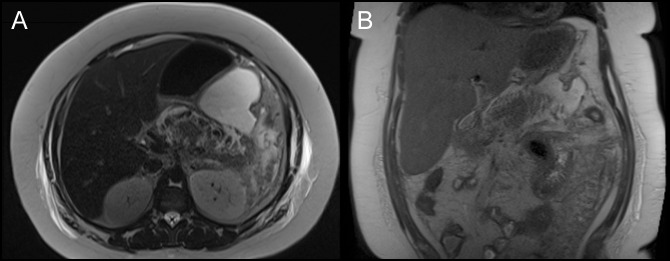
Magnetic resonance cholangiopancreatography in (A) axial and (B) coronal views obtained on day 10 of admission. Hemorrhagic and acute necrotizing pancreatic and peripancreatic fluid collections visualized, predominantly affecting body and tail of pancreas.

Her second episode of abdominal pain occurred several weeks later. On arrival, she was tachycardic but normotensive, and in no apparent distress. Initial laboratory evaluation was notable for leukocytosis to white blood cell count 20 K/μL with normal hemoglobin and comprehensive metabolic panel (including hepatic function panel) and lipase. Subsequent abdominal and pelvic CT demonstrated acute interstitial pancreatitis located predominantly within the body and tail (sparing the head), with surrounding stranding and without necrosis, fluid collections, or biliary or pancreatic ductal abnormalities (Figure 1). Extensive evaluation as to etiology was again performed and unremarkable for clear cause of pancreatitis. This included hereditary pancreatitis testing, which returned negative for *PRSS1*, *SPINK1*, *CTRC*, and *CFTR* genes. She was initiated on lactated Ringer's 3 cc/kg; however, over the initial 12 hours after presentation, she was noted to have elevated lactate to a peak of 6.3 mmol/L. She became hypoxemic with tachypnea necessitating intubation and transfer to the intensive care unit in the setting of hypoxic respiratory failure from severe acute respiratory distress syndrome. Her course was complicated by renal failure necessitating sustained low efficiency dialysis, shock bone marrow precipitating progressive anemia, thrombocytopenia, and diffuse ileus. After a 7-day intensive care unit course and transition to the medicine hospital ward, she experienced cyclical fevers to 40.2°C, prompting repeat abdominal imaging with magnetic resonance cholangiopancreatography on day 10 of admission. This demonstrated hemorrhagic pancreatitis with acute necrotizing fluid collections; however, no free air suggestive of infection (Figure 2). These fluid collections were deemed too early for endoscopic intervention. She continued to receive supportive care with pain control, fluid resuscitation, and early enteral feeds until improvement. She was gradually weaned from dialysis after kidney recovery postacute tubular necrosis injury and is planned for repeat imaging as an outpatient. After extensive chart review and negative aforementioned workup, it was deemed that her presentation was most consistent with liraglutide-induced hemorrhagic pancreatitis.

## DISCUSSION

We present a case of liraglutide-induced acute pancreatitis in a patient without diabetes. We suspect liraglutide as the culprit, given its temporal association to the patient's symptoms and exhaustive unremarkable evaluation for alternative etiologies of pancreatitis. Although the literature has highlighted the risk of liraglutide-induced pancreatitis in diabetic populations, particularly with concern for increased risk of gallstone disease, there remain inconclusive data whether liraglutide-induced pancreatitis has an association with pancreatitis independent of gallstone disease.^3–7^ In addition, it remains unclear whether weight loss formulations of liraglutide, which may be dosed nearly 3 times that of diabetic dosing (3.0 mg once daily vs 1.2 mg once daily, respectively), has an increased association with acute pancreatitis in the nondiabetic population. This case highlights the importance of recognizing this potential association in patients with obesity undergoing medical therapy for weight loss with GLP-1 agonists.

GLP-1 agonists have been theorized to increase the risk of acute pancreatitis through 2 primary mechanisms. Most commonly, owing to the drug's effect of decreased biliary and gallbladder motility, there has been an increased risk of gallstone formation demonstrated in these patients.^8,9^ However, there has been a correlation with the increased risk of acute pancreatitis and GLP-1 agonists even in the absence of gallstone formation, most likely related to GLP-1 effects on expansion of the exocrine pancreas. A rodent study showed increased ductal proliferation and metaplasia in rats with acute hemorrhagic pancreatitis exposed to GLP-1 agonists.^1^ Beyond rodent models, ductal proliferation and metaplasia have previously been established as well-recognized mechanisms for pancreatitis in humans.^10,11^ A final consideration is that patients exposed to GLP-1 agonists remain at risk for acute pancreatitis for as long as 2 years after drug discontinuation.^4^ Our patient discontinued Saxenda between 35 and 50 days before admission yet developed this severe complication.

Future study considerations should be focused on the risk of acute pancreatitis associated with GLP-1 use in patients without diabetes, as was the case with our patient. Further study with higher-dose formulations for weight loss therapy would assist in elucidating direct drug effect vs secondary effect from the drug's modification of the diabetes disease course.

## DISCLOSURES

Author contributions: RD Dolan wrote the manuscript, revised it for intellectual content, and is the article guarantor. AN Bazarbashi, A Lo, and BN Smith revised the manuscript for intellectual content.

Financial disclosure: None to report.

Informed consent was obtained for this case report.
